# Insect-associated molecules at the plant immune interface: recognition, manipulation and resistance design

**DOI:** 10.1007/s44307-026-00147-2

**Published:** 2026-11-13

**Authors:** Xin-Feng Wang, Ya-Xuan Wang, Yi-Ming Ou, Feng-Xiang Lai, Qi Wei, Jia-Chun He, Wei-Xia Wang, Jin-Li Zhang, Qiang Fu, Pin-Jun Wan

**Affiliations:** State Key Laboratory of Rice Biology and Breeding, China National Rice Research Institute, Hangzhou, 310006 Zhejiang China

**Keywords:** Plant–insect interaction, Herbivore-associated molecular pattern, Salivary effector, Nucleotide-binding leucine-rich repeat receptor, Pattern-recognition receptor, Autophagy, Host plant resistance

## Abstract

Feeding and oviposition expose plants to insect-associated proteins, peptides, metabolites, enzymes and small RNAs. Depending on the delivery route, these molecules encounter tissue surfaces, wounds, the apoplast, vascular cells or intracellular compartments. Plant responses to these molecules range from defense activation to signaling diversion and immune suppression. Interpretation becomes difficult when release route, function and evidence strength are treated as interchangeable. We organize current evidence in a two-dimensional recognition–manipulation framework that treats plant recognition and host manipulation as independent, potentially co-occurring properties. The framework connects extracellular herbivore-associated molecular pattern (HAMP) perception and nucleotide-binding leucine-rich repeat receptor (NLR)-mediated effector recognition to recurrent host control points, including jasmonate–salicylate crosstalk, redox control, vesicle trafficking, protein turnover, autophagy, plasmodesmal callose and transcriptional regulation. The BISP–BPH14–OsNBR1 module provides a well-resolved example: BISP suppresses basal defense, triggers genotype-specific resistance and is subsequently cleared to attenuate immune activation. Chewing and piercing-sucking insects define illustrative feeding-mode endpoints, whereas gallers, miners and borers provide additional insect interfaces; spider mites are included only as non-insect arthropod comparators. We evaluate six independent evidence domains: candidate discovery, natural delivery, molecular activity, receptor or target engagement, organismal phenotype and ecological or agronomic context. Signal termination, growth recovery and yield protection are assessed separately. This framework prioritizes mechanisms for translational testing while retaining provisional candidates without imposing a universal resistance architecture.

## Introduction

Plant–insect interactions are repeated molecular exchanges, not simple contests between tissue loss and chemical defense. Plants integrate endogenous damage signals with insect-derived molecules and, in many systems, microbial or viral cues. Chewing insects expose large wound surfaces, whereas piercing**-**sucking insects repeatedly probe living cells and vascular tissues. Delivery geometry determines which plant compartments encounter a signal, but it does not determine whether a molecule functions as an elicitor or an effector. The same secretion can contain enzymes that generate active ligands, proteins that suppress immunity and molecules that are indispensable for feeding yet immunogenic to the plant. The resulting complexity has moved the field from salivary-protein inventories toward mechanistic questions about perception, host targeting and context-dependent outcome (Erb and Reymond 2019; Howe and Jander 2008).

A central obstacle is that terminology often mixes molecular origin, release route and biological function. Saliva, oral secretions, regurgitant, frass and oviposition-associated material describe how molecules reach plant tissues. Herbivore-associated molecular patterns (HAMPs), elicitors and effectors instead describe inferred activities (Acevedo et al. 2015; Arimura 2021). A HAMP is most useful when it denotes an insect-associated cue whose perception produces a reproducible defense response. An elicitor can be broader, including endogenous plant fragments or enzymes that indirectly release active signals. Effector should be reserved for a molecule that alters a host process in a way that affects herbivore performance. The categories overlap because an effector may be recognized in a resistant genotype or an essential salivary component may activate immunity after natural delivery. Release route defines exposure context; functional class remains an independent empirical question. Pattern-triggered immunity (PTI) refers to defense initiated by perception of molecular patterns, usually through cell-surface receptors.

Evidence strength now matters as much as candidate discovery. Salivary-gland enrichment, a signal peptide or detection in an artificial diet provides discovery evidence, not functional closure. Transient expression can reveal molecular activity, but it may misrepresent concentration, timing or cellular location. Strong inference requires a chain from natural delivery to receptor or target engagement, genetically supported immune output and insect phenotype. Inceptin receptor studies provide this type of closure for extracellular recognition, whereas target-resolved effector studies increasingly connect molecular binding to herbivore performance (Steinbrenner et al. 2020). Evolutionary work further shows that receptor gain and loss, effector diversification and host-dependent expression can all change the same interaction (Snoeck et al. 2022a).

Mechanistic completeness and agricultural usefulness are related but not interchangeable. In rice, BPH14-interacting salivary protein (BISP), the intracellular nucleotide-binding leucine-rich repeat receptor (NLR) BPH14 and the selective-autophagy cargo receptor OsNBR1 connect recognition with removal of the eliciting molecule (Guo et al. 2023). This system demonstrates that defense duration can be regulated independently of initial activation, although its architecture is specific to the tested rice–planthopper interaction. A resistance-associated plant allele may nevertheless merit breeding evaluation before every insect countermeasure is known. Effector-triggered immunity (ETI) refers to defense initiated when an immune receptor detects an effector or its action; it should not be inferred from defense-marker induction alone.

Previous reviews have established the molecular basis of plant–herbivore interactions by synthesizing herbivore perception, hormone signaling, insect effectors and plant defense outputs (Arimura 2021; Erb and Reymond 2019). Crop-focused syntheses have traced the perception of HAMPs and damage-associated molecular patterns (DAMPs), which report tissue damage, downstream signaling and insect counter-defense in rice (Kuai and Lou 2024). Recent broader reviews have emphasized structural and chemical defenses, biotechnology, sustainable pest management and breeding application (Vasantha-Srinivasan et al. 2025; Zhu et al. 2026). Building on these contributions, this Review organizes insect-associated molecules along two independent axes—plant recognition and host manipulation—and evaluates each case through six non-cumulative evidence questions. This organization links spatial delivery and mechanistic closure to biological outcome, failure mode and resistance-strategy choice, thereby distinguishing promising candidates from mechanisms ready for crop-level testing.

### Scope and selection criteria

This selective critical narrative Review draws on PubMed and Web of Science records retrieved on 22 June 2026 using terms covering plants, herbivorous insects, effectors, elicitors or oral secretions, and plant immunity or defense. The combined exports yielded 1,251 records after deduplication and were supplemented with targeted literature additions. Studies published since 2000 were prioritized. Cases were selected for relevance to molecular origin, delivery, recognition or host manipulation and for addressing at least one evidence question in Table 3. Candidate-discovery studies are distinguished from functional evidence, and insect-internal functions without a demonstrated or proposed plant-interface role are excluded. Unresolved receptor, target and phenotype links are identified explicitly. The synthesis is not systematic or exhaustive and does not support estimates of mechanism prevalence.

**Fig. 1:**
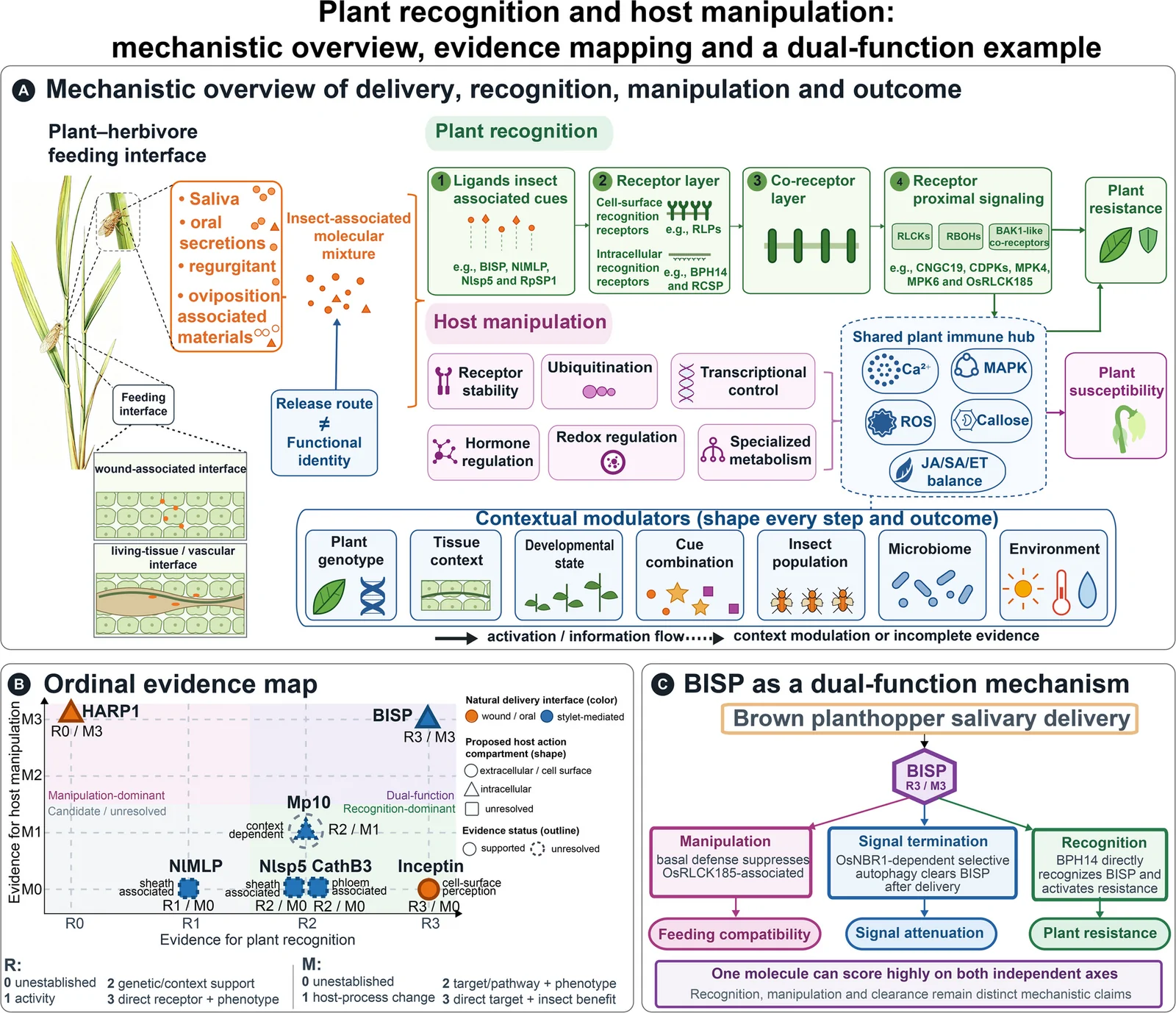
Plant recognition and host manipulation: mechanistic overview, evidence mapping and a dual-function example. **A** Insect-associated molecules delivered at wound-associated or living-tissue/vascular interfaces can be perceived by plant receptor complexes or manipulate host processes. Recognition and manipulation converge on shared immune hubs, and their effects on plant resistance, susceptibility and insect performance are conditioned by plant genotype, tissue context, developmental state, cue combination, insect population, microbiome and environment. Solid arrows indicate activation or information flow, whereas dashed arrows indicate contextual modulation or incomplete evidence. **B** Representative molecules are positioned independently according to the highest evidence level currently supported for plant recognition (R0–R3) and host manipulation (M0–M3). Coordinates indicate evidence closure rather than molecular potency or biological importance. Recognition evidence is defined as R0, unestablished; R1, defense-associated plant activity; R2, host-genetic or contextual support without a resolved direct receptor; and R3, direct receptor engagement with genetic and defense-phenotype support. Manipulation evidence is defined as M0, unestablished; M1, host-process change without resolved insect benefit; M2, target or pathway support linked to an insect phenotype; and M3, direct target engagement causally linked to insect benefit. The figure label “unestablished” means unestablished in the assessed context, not demonstrated absence. Coordinates summarize the cited assays and need not represent simultaneous activities in a single plant genotype. The recognition components in Panel A are a conceptual inventory: cell-surface receptors and intracellular NLRs are alternative recognition routes, not successive components of one membrane complex. Orange and blue denote wound/oral and stylet-mediated natural delivery, respectively; circles, triangles and squares denote extracellular or cell-surface, intracellular and unresolved proposed host action compartments, respectively. Short labels indicate tissue-level deposition contexts, such as sheath-associated or phloem-associated delivery, rather than host action compartments. Solid outlines indicate supported compartment assignment and natural-host context, whereas dashed outlines indicate that localization or natural-host evidence remains unresolved. **C** BISP suppresses basal defense associated with OsRLCK185, is directly recognized by BPH14, and is subsequently cleared through OsNBR1-dependent selective autophagy, attenuating immune activation. BAK1, Brassinosteroid insensitive 1-associated kinase 1; BISP, BPH14-interacting salivary protein; BPH14, brown planthopper resistance protein 14; Ca^2+^, calcium ion; CDPK, calcium-dependent protein kinase; CNGC19, cyclic nucleotide-gated channel 19; ET, ethylene; HAMP, herbivore-associated molecular pattern; JA, jasmonic acid; LecRK, lectin receptor kinase; MAPK, mitogen-activated protein kinase; NlMLP, *Nilaparvata lugens* mucin-like protein; OsNBR1, rice NBR1 selective-autophagy cargo receptor; OsRLCK185, rice receptor-like cytoplasmic kinase 185; RBOH, respiratory burst oxidase homolog; RCSP, a Toll/interleukin-1 receptor-type nucleotide-binding leucine-rich repeat receptor recognizing salivary chemosensory proteins; RLCK, receptor-like cytoplasmic kinase; RLP, receptor-like protein; ROS, reactive oxygen species; SA, salicylic acid. CathB3, aphid cathepsin B3; HARP1, cotton bollworm oral-secreted effector; INR, inceptin receptor; JAZ, jasmonate ZIM-domain; Mp10, green peach aphid chemosensory effector protein; NLR, nucleotide-binding leucine-rich repeat receptor; Nlsp5, planthopper salivary protein 5; RpSP1, a pentatomomorphan salivary protein

Rice–planthopper and selected solanaceous interactions receive particular attention because they offer complementary examples of receptor or target engagement, host genetics and insect phenotypes. This emphasis reflects case selection, not a comparative demonstration that other taxa have less complete mechanisms. Aphids, whiteflies, other hemipterans and the Hessian fly provide contrasts in feeding interface and host adaptation. Beetle-associated microbial manipulation is represented by the Colorado potato beetle example discussed below (Chung et al. 2013). Thrips are not covered as a separate case series; this is a coverage limitation rather than evidence that they lack relevant molecules. Plant-parasitic nematodes fall outside the selected arthropod focus. Spider mites are arachnids, not insects, and are included as cross-arthropod comparators of salivary activity and host-responsive expression. Microbial and viral examples are included only where they clarify molecular origin, indirect elicitation or the distinction between herbivore and pathogen benefit. Their encoding organism is kept separate from the insect delivery context. Figure 1 and Tables 1, 2 and 3 organize these selected examples by their supported mechanisms and unresolved links.

**Table 1:** Representative recognition and resistance modules. Entries distinguish demonstrated findings from the claim still requiring evidence, using Table 3. They are not cumulative confidence ranks. Endogenous plant cues and mite comparators are identified explicitly. An unresolved item is not evidence of biological absence; ecological generality and post-attack yield recovery are not established by the assays summarized unless stated. HAMP, herbivore-associated molecular pattern; ROS, reactive oxygen species

Signal / molecule	Origin / system	Plant sensor or implicated pathway	Supported evidence; limiting inference	Reference
Inceptin	Caterpillar oral secretion; legumes	INCEPTIN RECEPTOR (INR) receptor-like protein	Natural ligand delivery, INR genetics and antiherbivore responses. Limits: crop-network compatibility and field persistence; insect-gene perturbation is not an origin requirement for this plant-derived ligand	(Steinbrenner et al. 2020)
Inceptin receptor variation	Legume lineages	Gain or loss of INCEPTIN RECEPTOR (INR)	Receptor gain/loss explains lineage responsiveness. Limits: sensitivity is not equivalent to yield protection or population-wide resistance	(Snoeck et al. 2022a)
BISP	Brown planthopper–rice	BPH14 nucleotide-binding leucine-rich repeat receptor; OsRLCK185; OsNBR1	Direct recognition and OsNBR1-dependent clearance; persistent activation increases resistance but can carry growth/yield costs. Limits: optimal timing under repeated attack and population durability	(Guo et al. 2023)
NlCSP11; aphid Mp10	Distinct planthopper and aphid CSPs tested in Nicotiana	RCSP Toll/interleukin-1 receptor NLR	RCSP-dependent ectopic responses and NlCSP11 interaction evidence. Limits: native exposure and resistance must be assessed separately for each host–insect pair; these proteins are not aliases	(Rao et al. 2025)
OsPep3	Rice endogenous peptide	PEPR signaling	Plant peptide/receptor signaling and resistance assays. Limits: natural feeding-induced dose and agricultural delivery; insect-encoded origin is not applicable to this DAMP	(Shen et al. 2022)
Extracellular ATP (eATP)	Damaged plant tissue	DORN1–CNGC19 signaling	Genetic support for eATP–DORN1–CNGC19 signaling during herbivory. Limits: this is damaged-self perception, not identification of an insect ligand	(Kundu et al. 2025a)
Nlsp5	Brown planthopper–rice	Receptor unresolved	Sheath function, insect perturbation and elicitor activity. Limits: plant sensor and separation of structural feeding defects from immune-mediated effects	(Qi et al. 2025)
NlMLP	Planthopper–plant	Receptor unresolved	Feeding/sheath requirement and immune activation. Limits: receptor identity and whether immune activity explains the knockdown feeding phenotype	(Shangguan et al. 2018)
NlG14	Brown planthopper	Receptor unresolved	Plant defense activation. Limits: native effective dose, sensor identity and causal contribution during natural feeding	(Gao et al. 2022)
TePDI / conserved PDI	Spider mite–solanaceous plant comparator	Immune-signaling dependence; sensor unresolved	TePDI-associated defense activation and arthropod conservation. Limits: sensor identity, native exposure and suppression by co-delivered effectors	(Cui et al. 2023)
Aphid CathB3	*Myzus persicae* lineages–tobacco	EDR1-like cytoplasmic kinase	Propeptide–EDR1-like binding, phloem ROS and improved insect performance after CathB3 knockdown. Limits: adaptation-associated delivery differences and broader host applicability	(Guo et al. 2020)
Te16/spider-mite elicitors	Mite–plant	Calcium and BRI1-ASSOCIATED RECEPTOR KINASE 1-associated signaling	ROS, callose and JA-associated responses. Limits: direct sensor and causal interpretation in each natural mite–host pair	(Cui et al. 2024)
RpSP1-like protein	Pentatomomorphan saliva	HSP40-associated	Defense activation and feeding requirement. Limits: whether immune alteration, rather than another feeding function, causes the insect phenotype	(Hu et al. 2025)

### Operationalizing the recognition–manipulation map

Figure 1B uses two independent ordinal evidence axes, not quantitative measurements of recognition or manipulation strength. Recognition levels distinguish an unestablished recognition mechanism in the assessed context (R0), defense-associated plant activity (R1), activity supported by host genetics or context-dependent response evidence without a resolved direct receptor (R2), and direct receptor engagement linked through genetic evidence to a defense phenotype (R3). Manipulation levels distinguish no established manipulation in the assessed context (M0), alteration of a host process without a resolved link to insect benefit (M1), pathway or target-associated activity linked to an insect phenotype (M2), and direct target engagement causally linked to insect benefit (M3). Thus, R0 and M0 do not mean biological absence, and M1 does not establish adaptive manipulation. Table 3 supplies independent questions for auditing each assignment; its rows are not added to calculate a coordinate. Delivery, molecular activity, engagement and phenotype must be considered in their respective assay contexts, while ecological evidence delimits generality rather than automatically increasing a score. Figure 1B illustrates HARP1 (R0/M3), BISP (R3/M3), Mp10 (R2/M1), NlMLP (R1/M0), Nlsp5 (R2/M0), CathB3 (R2/M0) and inceptin (R3/M0), with the associated evidence and limitations discussed in Tables 1 and 2 and the molecule-specific text. Each position is a qualitative synthesis across the cited assays, not a claim that both coordinates were measured simultaneously in one genotype or feeding experiment. BISP recognition, for example, requires the appropriate BPH14 background; its manipulation and recognition properties should not be assumed to have identical outcomes in all rice genotypes. The Fig. 1C mechanism illustrates this distinction. Neither axis measures potency, abundance, field durability or technological readiness.

**Table 2:** Evidence profiles for host manipulation and its consequences. Findings and limits are recorded by molecule rather than collapsed into a cumulative grade. Pathway dependence is distinguished from direct targeting, and insect-essentiality phenotypes do not alone prove host-mediated virulence. Mite and virus comparators are identified. Field durability and post-attack yield recovery require evaluation beyond the mechanistic assays summarized. DAMP, damage-associated molecular pattern; ER, endoplasmic reticulum; JA, jasmonic acid; JAZ, JASMONATE ZIM-DOMAIN; RNAi, RNA interference; SA, salicylic acid

Molecule / candidate	Origin / system	Direct plant target or implicated pathway	Supported evidence; limiting inference	Functional designation / consequence	Reference
HARP1	Cotton bollworm	JAZ repressors	JAZ interaction and mechanism with plant/insect phenotypes. Limits: activity across JAZ paralogs, natural-dose comparisons and transfer to crop backgrounds	Effector justified; blocks COI1-dependent JAZ degradation	(Chen et al. 2019)
BISP	Brown planthopper	OsRLCK185/BPH14/OsNBR1	OsNBR1 loss slows clearance and increases resistance during prolonged attack; constitutive BISP causes growth/yield costs. Limits: net yield benefit of altered natural clearance	Suppresses basal defense; recognized by BPH14; clearance attenuates stimulation	(Guo et al. 2023)
Al106	*Apolygus lucorum*	PUB33	PUB33 interaction, altered ubiquitination and feeding phenotypes. Limits: a selective plant edit retaining endogenous PUB33 function remains to be established	Effector justified; interferes with ubiquitination and promotes feeding	(Dong et al. 2023)
NlVRSP1	Planthopper	OsPDCB1	OsPDCB1 interaction, callose/feeding phenotypes and resistance-associated haplotype introgression. Limits: selective binding escape versus enhanced host function, transport costs and durability	Effector justified; disrupts plasmodesmal callose defense	(Yang et al. 2026)
Mp10	Green peach aphid	EDS1-associated DAMP signaling; direct target unresolved	Induced Mp10 increases baseline resistance but removes extra OG protection in wild type; OG protection returns in eds1 × Mp10. Limits: mechanism and native dose	Suppression and activation depend on host background and endpoint	(Gravino et al. 2025)
ApMIF family; MpMIF1	Pea aphid; green peach aphid–Nicotiana	Immune pathway target unresolved	Diet secretion and plant-specific RNAi phenotypes; MpMIF1 plant-expression rescue. Limits: ApMIF1–3 co-silencing, host target identity and native plant localization	MIF host-dependent function supported; species-and family-level evidence must remain distinct	(Naessens et al. 2015)
Armet	Pea aphid	Salicylic-acid metabolism/signaling; direct target unresolved	Delivery and insect RNAi phenotypes; plant SA/pathogen responses. Limits: the measured plant response did not improve aphid performance in the tested assays	Feeding requirement and plant activity demonstrated; their causal linkage is unresolved	(Wang et al., 2015a); (Cui et al. 2019)
Me10	Aphid	TFT7	Me10–TFT7 interaction and host genetic evidence. Limits: native exposure, insect-side specificity and generality across aphid–host combinations	Effector supported; links 14–3-3 signaling to non-host resistance	(Chaudhary et al. 2019)
VPS52-targeting aphid effector	Aphid	VPS52	VPS52 interaction and host-dependent plant phenotypes. Limits: preserved endogenous trafficking and native-dose validation of target-directed intervention	Effector supported; host-specific trafficking manipulation	(Rodriguez et al. 2017)
Bt56	Whitefly	NTH202/salicylic-acid signaling	Salivary delivery, NTH202 interaction, plant perturbation and insect performance evidence. Limits: full interaction-to-hormone causality and generality across hosts	Target-associated effector; SA-linked susceptibility in the tested system	(Xu et al. 2019)
BtFer1	Whitefly	Redox and callose pathways; direct target unresolved	Ferritin biochemical activity with redox/callose and host-dependent feeding effects. Limits: relevant in vivo substrate context and transferability, not necessarily a protein receptor	Effector supported by delivery and phenotype; suppresses H2O2 and callose in a plant-dependent manner	(Su et al. 2019)
Bsp9	Whitefly	WRKY33–MPK6	WRKY33 binding and MPK6-complex interference with insect/vector assays. Limits: contribution relative to other effectors under matched natural exposure	Effector justified; suppresses immunity and supports vector performance	(Wang et al. 2019)
Sm9723	Grain aphid–wheat; activity assays in *N. benthamiana*	Defense pathway; direct target unresolved	Immune suppression in *N. benthamiana*; insect RNAi phenotypes on wheat. Limits: these assays do not establish a wheat immune mechanism or its direct target	Candidate immune effector; activity and insect performance demonstrated in different plant systems	(Zhang et al. 2022)
Salivary DNase II	Planthopper	Defense pathway; direct host target unresolved	Enzymatic activity and insect perturbation evidence. Limits: the causal host substrate/pathway and its contribution during natural feeding	Effector supported; suppresses rice defense	(Huang et al. 2019)
NtRLP4-destabilizing effectors	Multiple herbivores	NtRLP4	Effector-associated NtRLP4 destabilization and host susceptibility. Limits: distinguish individual effector evidence from class-wide generalization and establish crop applicability	Effector class supported; convergent receptor destabilization	(Wang et al., 2026b)
PPI5	Cotton bollworm	GhFKBP17-2	GhFKBP17-2 interaction and biochemical/genetic evidence. Limits: selective protection that retains host protein-folding functions and avoids growth costs	Effector justified; suppresses ER immunity and JA/SA defense	(Wang et al. 2024b)
HYPB1	Cotton bollworm	GhDIR15	GhDIR15 interaction and plant/insect phenotypes. Limits: specificity of the metabolic effect, natural exposure and agronomic trade-offs	Effector justified; reduces specialized-metabolite biosynthesis	(Wang et al., 2026c)
CMV 2 b	Cucumber mosaic virus in an aphid-vector system	JAZ proteins	JAZ interaction and vector-attraction effects in a virus–plant–vector system. Limits: virus benefit is not equivalent to vector benefit; not insect-derived	Virus-encoded comparator; prevents JAZ degradation and increases vector attraction	(Wu et al. 2017)
BtmiR29-b; miR-7-5P	Whitefly–tobacco; planthopper–rice	NtBAG4 via AGO1; OsbZIP43 and other transcripts	BtmiR29-b: salivary transfer and AGO1-associated targeting. miR-7-5P: host-dependent insect phenotype, plant rescue and target-site tests. Limits: native dose, additional targets and cross-pair generality	Molecule-specific RNA-effector evidence; not a uniformly provisional class	(Han et al. 2025; Zhang et al. 2024)
SHOT family	Spider-mite comparator	Targets unresolved	Secretome and host-responsive expression evidence. Limits: individual activity, host targets and causal performance effects; plasticity alone does not establish adaptation	Candidate family only; host-responsive secretion plasticity	(Jonckheere et al. 2018)

## Separating release route, immune function and evidence strength

The feeding interface is a chemically heterogeneous reaction zone, so the active signal is not always the molecule first detected in an insect secretion. Caterpillar oral secretions contain fatty acid–amino acid conjugates, enzymes, peptides and microbe-associated products, while stylet feeders alternate gelling and watery saliva during pathway and ingestion phases. Plant material is simultaneously ruptured, oxidized and enzymatically modified. Consequently, the biologically active entity may be an insect molecule, a plant fragment released by an insect enzyme or a composite signal produced only in damaged tissue. Classical work showed that fatty acid–amino acid conjugates in *Manduca sexta* oral secretions are necessary and sufficient for herbivore-specific responses in *Nicotiana attenuata* (Halitschke et al. 2001). Activity-mapping approaches later demonstrated that different fractions of herbivore secretions engage distinct hormone outputs (Schmelz et al. 2009).

Damaged-self signaling supplies a parallel information channel. DAMPs, including extracellular ATP, cell-wall fragments and endogenous peptides, amplify local damage into coordinated immunity. Plant elicitor peptides are conserved signals that regulate both direct and indirect antiherbivore defense, and the *Arabidopsis* Pep–PEPR system contributes to jasmonate-dependent resistance after feeding (Huffaker et al. 2013; Klauser et al. 2015). In rice, OsPep3 signaling extends this logic to piercing**-**sucking insects and pathogens (Shen et al. 2022). The DAMP evidence makes it unsafe to attribute every feeding-induced response to a salivary ligand. A defensible interpretation requires wound-only controls, inactive molecular variants and plant mutants that separate the insect-derived cue from downstream damage amplification.

Plant genotype determines whether a delivered signal becomes immunologically visible. Inceptin perception depends on a lineage-restricted receptor-like protein, and evolutionary gain or loss of that receptor explains much of the variation in responsiveness among legumes. A comparable principle applies to rice receptor kinases, where OsLRR-RLK1 contributes to early defense against a chewing herbivore without establishing that it directly binds an insect ligand (Hu et al. 2018). Co-receptor and kinase dependence are informative, but neither establishes receptor identity. This distinction is especially important for BAK1/SERK3, which participates in many immune complexes and affects herbivore-induced jasmonate accumulation but is rarely sufficient to establish direct HAMP recognition (Yang et al. 2011).

Feeding necessity and immune manipulation must also be separated. RpSP1-like proteins and NlMLP activate plant defenses while contributing to insect feeding or sheath formation (Hu et al. 2025). For NlMLP, defective sheath construction provides an alternative explanation for reduced feeding after knockdown (Shangguan et al. 2018). These observations support immunological visibility and a feeding function, but do not by themselves show that alteration of plant immunity causes the insect phenotype. A decisive comparison would retain secretion and sheath function while changing immune activity, or ask whether the feeding defect is rescued on a plant lacking the relevant immune response. Essentiality may constrain escape, but functional redundancy, altered expression or reduced exposure could still permit evasion. Such proteins are promising targets because of a testable constraint, not because essentiality guarantees durable resistance.

Complex secretions also create indirect elicitation. Gut-associated bacteria deposited during herbivory can trigger plant responses, and in some systems insects exploit bacteria to suppress rather than activate defense (Chung et al. 2013). Parasitoid-associated polydnaviruses can alter caterpillar physiology and, indirectly, the plant response to feeding (Tan et al. 2018). Frass adds another layer: maize chitinases ingested and excreted by fall armyworm can suppress herbivore defenses, demonstrating that plant proteins may be repurposed during passage through the insect (Ray et al. 2016). Credible assignment of activity to a purified molecule first requires experiments on the complete biological unit, including insect microbiota and natural secretions.

Secretome studies reveal diversity but also expose a sampling problem. Spider-mite saliva contains expanded families of proteins with unknown functions (Jonckheere et al. 2016). SHOT-family transcripts change rapidly after host transfer and show asymmetric reversal across generations (Jonckheere et al. 2018). These dynamics support host-responsive deployment; they do not distinguish an adaptive effector response from altered nutrition, stress or a change in the cells contributing RNA. Nor does failure to detect a low-abundance protein establish that it is not delivered. Common-garden rearing, reciprocal host transfers and protein measurements during feeding can separate stable lineage differences from reversible expression states. Individual perturbations then test whether the response benefits the insect through a plant target. Before that step, SHOT proteins remain candidates, and their expression plasticity cannot be counted as evidence of immune suppression.

## Spatial delivery constrains molecular opportunity

Natural delivery is the first mechanistic gate because a molecule can act only in a compartment that it reaches at a relevant dose. Aphid effectors provide unusually clear spatial evidence. Mp10 accumulates in the cytoplasm and chloroplasts of mesophyll cells adjacent to the stylet path, whereas MpPIntO1 and MpC002 are associated mainly with the stylet sheath (Mugford et al. 2016). The observed distribution indicates a division of labor between molecules that remain extracellular or structural and molecules that enter living plant cells. The same spatial restriction explains why whole-leaf overexpression can produce misleading phenotypes: it abolishes the steep spatial gradients created by localized salivation and exposes cell types that may never encounter the effector during feeding.

Effector access can itself be a point of resistance. A caterpillar effector enters plant cells through endocytosis, and jasmonate opposes this process (Yan et al. 2023). This observation distinguishes two explanations for a weak phenotype: insufficient delivery to the target compartment, or insufficient activity after arrival. Intracellular expression bypasses the first limitation and therefore cannot test the complete natural mechanism. Matched extracellular application and intracellular expression, with uptake measurements and membrane-integrity controls, could distinguish them. If the phenotype disappears only when extracellular access is restricted, entry is limiting; if both routes fail in a target allele, engagement or downstream signaling is limiting. These alternatives lead to different interventions, even when their endpoint defense markers look similar.

Piercing**-**sucking insects create a temporally structured interface. Gelling saliva forms a sheath along the stylet pathway, whereas watery saliva is delivered in brief intracellular punctures and during sustained ingestion. Nlsp5 illustrates how these functions can intersect: it is required for salivary sheath formation and also acts as a HAMP that induces plant resistance (Qi et al. 2025). NlMLP similarly contributes to successful feeding while exposing the insect to immune surveillance (Shangguan et al. 2018). Feeding phase changes the tissues exposed to saliva: pathway activities can expose epidermal and mesophyll cells, whereas sustained phloem feeding exposes sieve-element-associated compartments. This anatomical distinction does not by itself identify any particular protein as an early- or late-delivered effector; such assignments require molecule-specific detection linked to feeding phase.

Subcellular localization can reveal the host process being targeted. HARP1 enters the nucleus and binds jasmonate ZIM-domain (JAZ) repressors, thereby stabilizing them against COI1-dependent degradation (Zhang et al., 2026a). PPI5 localizes to the endoplasmic reticulum and targets GhFKBP17-2, linking an insect effector to protein-folding stress and defense regulated by the phytohormones jasmonic acid and salicylic acid (JA and SA) (Wang et al. 2024c). Aphid Me10 interacts with the tomato 14–3-3 isoform TFT7, whereas an aphid effector targeting VPS52 implicates endomembrane trafficking (Chaudhary et al. 2019; Rodriguez et al. 2017). Localization and interaction data are most informative in combination. Localization narrows the plausible mechanism; direct interaction and genetic dependence distinguish functional targeting from co-occupancy.

Secreted proteins can expose the insect to defense as well as facilitate feeding. Whitefly ferritin alters redox-associated defenses, whereas aphid CathB3 activates phloem defense through its propeptide interaction with the host EDR1-like kinase (Guo et al. 2020; Su et al. 2019). Conserved TePDI from the spider mite *Tetranychus evansi* triggers immunity in solanaceous plants, but other mite effectors suppress its cell-death activity (Cui et al. 2023). This comparator demonstrates that an immunogenic protein can be retained within a repertoire that counteracts its recognition-associated effects. Purified-protein activity and the response to complete saliva can therefore differ without either observation being erroneous. The active processed form, access to the host partner and co-delivered suppressors must be distinguished; secretion alone does not establish where the immune interaction occurs.

Exposure also has a time dimension. Selective effector clearance can remove the stimulus for recognition after feeding ends (Guo et al. 2023), but declining defense markers could alternatively reflect receptor desensitization, tissue damage or exhaustion of a downstream response. Withdrawal and re-challenge experiments distinguish these possibilities: a recovered tissue should retain the capacity to respond to renewed attack. Ligand abundance, receptor activity and growth should therefore be followed separately. Spatial delivery identifies which interactions are possible; temporal measurements determine whether those interactions persist long enough to influence feeding and whether they cease without disabling subsequent defense.

## Extracellular recognition and intracellular surveillance

Extracellular recognition is best resolved when both the active molecule and its signaling-competent receptor complex are defined. Inceptins are proteolytic fragments of the chloroplastic ATP synthase γ-subunit generated in caterpillar guts. They return to damaged leaves in oral secretions and provide an early example of herbivore-associated elicitors derived from ingested plant proteins (Schmelz et al. 2006). Subsequent work identified INCEPTIN RECEPTOR (INR) as a lineage-restricted legume leucine-rich repeat receptor-like protein that directly mediates inceptin perception but lacks a cytoplasmic kinase domain (Steinbrenner et al. 2020). INR constitutively associates with the adaptor receptor kinase SUPPRESSOR OF BIR1-1 (SOBIR1) and recruits SOMATIC EMBRYOGENESIS RECEPTOR KINASE (SERK) family co-receptors in a ligand-dependent manner. This signaling-competent complex closes the causal chain from ligand binding through immune activation to antiherbivore defense. Comparative genomics further indicates that gain and loss of functional INR homologs explain lineage-specific differences in inceptin sensitivity (Snoeck et al. 2022a). Table 1 compares this level of closure with modules for which the receptor, target or ecological output remains unresolved.

Recognition can also scale beyond direct defense. In common bean, INR-mediated perception promotes the emission of a distinctive volatile blend that recruits predatory wasps, directly connecting molecular recognition to tritrophic interactions (Guayazán Palacios et al. 2026a). In cowpea, the circadian clock gated the INR-mediated transcriptional response. Identical In11 treatments elicited stronger late-phase defense-gene expression during the day than at night, and this temporal dependence was lost in arrhythmic plants (Guayazán Palacios et al. 2026b). The two studies extend receptor–phenotype analysis beyond larval growth to time-dependent transcriptional outputs and community-level consequences. They expose an agronomic constraint: transferring a receptor alone may not reproduce the desired resistance phenotype unless the recipient crop provides compatible co-receptors, downstream signaling components, metabolic capacity and ecological partners.

Plants also perceive endogenous signals generated by feeding. The DORN1 extracellular ATP receptor and the CNGC19 calcium channel act in tandem during Spodoptera litura herbivory, integrating damaged-self perception with calcium influx (Kundu et al. 2025a). Herbivore-derived fatty-acid amides can trigger a reactive oxygen burst, whereas different oral-secretion components produce distinguishable early signaling profiles (Block et al. 2018). Convergence on calcium and reactive oxygen species (ROS) does not imply a common receptor. Signal identity may instead be encoded at these shared decision nodes through amplitude, duration, subcellular origin and interaction with hormone pathways.

Receptor-like kinases can also tune the response to suppression. *N. attenuata* lectin receptor kinase 1 counteracts insect-mediated inhibition of induced defense during *M. sexta* herbivory (Gilardoni et al. 2011). In potato aphid interactions, an effector alters the conformation of a plasma-membrane somatic embryogenesis receptor kinase complex (Peng et al. 2016). The evidence supports receptor-proximal manipulation even when the ligand–receptor pair is incomplete. In the absence of physical ligand binding, receptor-complex dependence or remodeling is the more accurate description; direct recognition remains unproven.

Intracellular recognition provides a useful contrast between natural and reconstructed interactions. BPH14 directly binds BISP in the rice–brown planthopper system and activates resistance (Guo et al. 2023). In *Nicotiana benthamiana*, the Toll/interleukin-1 receptor-type NLR RCSP mediates responses to chemosensory proteins, including planthopper NlCSP11 and the distinct aphid protein Mp10 (Rao et al. 2025). Ectopic NlCSP11 expression, RCSP-dependent phenotypes and interaction assays support a recognition mechanism in the test plant; pathogen-directed systemic resistance is not equivalent to resistance against the source planthopper. Native delivery, effective dose and insect-side perturbation must be assessed for each natural host–insect pairing. RCSP thus expands the repertoire of possible surveillance mechanisms without establishing that NlCSP11 is naturally monitored by an equivalent receptor in rice.

Hessian fly–wheat interactions provide complementary population genetics. A massive expansion of candidate effector genes accompanies gall formation, and mapping of avirulence loci has begun to connect insect alleles with wheat resistance genes (Aggarwal et al. 2014; Zhao et al. 2015). The systems closely resemble classical gene-for-gene relationships, yet the causal chain from secreted protein to specific plant receptor remains incomplete for many loci. Population data from these systems reveal how virulence alleles spread after resistance deployment and how quickly an apparently effective *R* gene can be eroded at the population level.

Bt56 shows why a defense-associated response must be separated from its insect outcome. Bt56 expression promoted SA accumulation and whitefly performance, and the protein interacted with tobacco NTH202 (Xu et al. 2019). However, Bt56 expression did not significantly change the measured JA, jasmonoyl-L-isoleucine or JA-marker outputs; NTH202 silencing in uninfested plants also did not reproduce the same SA increase. These results support SA-associated susceptibility and a host interaction, but not a fully demonstrated linear NTH202**–**SA**–**JA suppression mechanism. Viral manipulation of hormone perception provides a separate route to vector attraction (Wu et al. 2017). In both cases, pathway activation must be interpreted against feeding or transmission outcomes rather than treated as evidence of protection.

These cases do not yet establish a universal hierarchy in which extracellular perception necessarily precedes or reinforces NLR recognition (Wu and Baldwin 2009). Shared calcium, ROS or hormone outputs can result from coupled pathways, parallel pathways or one pathway compensating for another. A stronger phenotype in a receptor combination is therefore insufficient to infer mechanistic coupling. Single and combined receptor perturbations, challenged with the same insect population and matched ligand exposure, should test whether the combined response departs from an explicitly defined additive expectation. The distinction matters for breeding: parallel barriers may retain protection after one is bypassed, whereas two receptors dependent on the same vulnerable signaling component can fail together.

## Effector targets reveal recurring vulnerabilities in plant immunity

Downstream of perception, JA signaling is a recurrent target because it coordinates antiherbivore metabolites, proteins and volatiles. Cotton bollworm HARP1 binds multiple JAZ repressors and prevents their COI1-dependent degradation, keeping defense transcription suppressed (Chen et al. 2019). Structural analysis has clarified how HARP1 reinforces JAZ-mediated repression (Zhang et al., 2026a). Plant or microbial factors can also activate jasmonate through JAZ destabilization, so the adaptive direction depends on feeding guild and ecological context. Table 2 compares target-resolved interactions with molecules supported only by pathway or phenotype evidence. A convincing effector study identifies the affected JAZ members, plant cell types and downstream outputs; a generic change in jasmonate-responsive genes is insufficient.

Whitefly effectors illustrate why convergence in insect performance need not imply identical host mechanisms. Bt56 engages NTH202 and promotes SA-associated susceptibility, whereas BtFer1 alters redox and callose-associated defenses (Su et al. 2019; Xu et al. 2019). Bsp9 interacts with WRKY33 and interferes with its association with MPK6 (Wang et al. 2019). These studies use different host and assay contexts, so their outcomes cannot be used to rank hormone diversion, redox control and transcriptional regulation as independent contributions to one natural infestation. Such a ranking would require matched single and combined perturbations. If two effectors act through the same limiting output, removing both may add little protection; if their functions compensate for one another, single-effector tests may underestimate the vulnerability of the combined system.

Mp10 separates loss of an inducible response from overall susceptibility. In wild-type *Arabidopsis* backgrounds, induced Mp10 expression increased baseline resistance to aphids, yet oligogalacturonide (OG) treatment no longer added protection. OG-induced protection was absent in plants lacking ENHANCED DISEASE SUSCEPTIBILITY 1 (EDS1), but reappeared in the corresponding Mp10-expressing background (Gravino et al. 2025). Thus, suppression of one elicitor response did not imply lower overall resistance, and restoration of OG responsiveness did not by itself establish a more resistant baseline state. This distinction is more informative than assigning Mp10 a fixed elicitor or suppressor label. EDS1 dependence does not establish direct binding; compensatory signaling and surveillance of disturbed receptor homeostasis remain alternative explanations. Because these comparisons use induced expression, their mechanistic interpretation should not be extended to native protein dose without independent delivery measurements.

Vesicle trafficking is a host-specific vulnerability. An aphid effector targets VPS52 in a manner that differs among host plants, and phloem-specific overexpression of VPS52 reduces aphid performance (Rodriguez et al. 2017). Me10 interacts with tomato TFT7, a 14–3-3 isoform required for resistance to non-adapted aphids (Chaudhary et al. 2019). The target data indicate that compatibility can be controlled by broadly conserved cellular proteins whose contribution becomes visible only in particular host–aphid combinations. A plausible route to host specialization is selection at the effector–target interface without wholesale rewiring of downstream immunity.

Protein turnover appears repeatedly among resolved targets, but two interpretations remain possible. Al106 perturbs PUB33-associated ubiquitination, and multiple herbivore effectors destabilize NtRLP4 (Dong et al. 2023; Wang et al., 2026b). These findings support convergence on particular host proteins or processes. SISP stabilized OsSR45, whereas NlOBP1b acted on the OsCK2 complex (Gao et al. 2026; Wang et al., 2026a). Together, these findings show that susceptibility can result from increased abundance or activity of a host regulator, not only from its depletion. However, recurrence in published studies may also reflect interaction-screen biases toward abundant proteins and familiar immune pathways. Demonstrating an evolutionarily preferred vulnerability requires comparing its frequency with an appropriate background of tested targets and confirming independent functional effects. A shared target is a reason to test broader protection, not evidence that editing it will provide broad-spectrum resistance.

Endoplasmic reticulum and protein-quality-control pathways connect secretion stress to immunity. Cotton bollworm PPI5 targets GhFKBP17-2 and inhibits its peptidyl-prolyl isomerase activity, weakening endoplasmic reticulum-associated immunity together with JA and SA responses (Wang et al. 2024c). Because secretory defense proteins and receptors depend on endoplasmic reticulum folding, local disruption can have system-wide consequences. The same mechanism links cellular homeostasis to herbivore susceptibility. Plant alleles that preserve client folding while reducing effector binding could enhance resistance without constitutive defense activation.

OsPDCB1 provides a more specific vascular example. Its X8 domain anchors callose, whereas the planthopper effector NlVRSP1 binds OsPDCB1 and disrupts callose-related defense. A resistance-associated OsPDCB1 haplotype improves resistance after introgression into susceptible japonica backgrounds (Yang et al. 2026). This links a defined host process to usable allelic variation, but two translational claims must remain distinct: enhancing callose-based protection and preventing effector binding. The haplotype result alone does not prove that resistance arises through selective loss of NlVRSP1 engagement. Allele-matched binding assays and complementation can resolve that distinction. Because plasmodesmal permeability also supports intercellular transport, resistance should be evaluated alongside assimilate movement and yield. More callose is not automatically a better agricultural outcome.

Selective autophagy exposes a timing trade-off rather than a general instruction to increase degradation. In the BISP system, removing the recognized effector attenuates immune stimulation (Guo et al. 2023). Earlier clearance could reduce harmful manipulation but also shorten recognition, whereas slower clearance could prolong protection and its growth cost. Which effect dominates should depend on the rate of delivery and the interval between attacks. We therefore predict that changing cargo removal will have different outcomes under a single feeding bout and repeated infestation. This prediction requires simultaneous measurements of effector persistence, resistance and post-attack growth; pathway activation alone cannot establish that autophagy is beneficial.

Small-RNA studies now provide molecule-specific evidence that can be compared with protein-effector mechanisms. Whitefly BtmiR29-b entered tobacco through salivary exosomes and used plant ARGONAUTE 1 (AGO1) to silence NtBAG4 (Han et al. 2025). In the rice–planthopper system, miR-7-5P silencing impaired feeding on plants but not on artificial diet, and plant expression of the miRNA rescued that defect. Target-site mutations in OsbZIP43 protected its immune activity from miRNA suppression (Zhang et al. 2024). These are substantive delivery, targeting and host-dependent phenotype data. Remaining questions concern effective native dose, additional targets and transferability across host–insect pairs, not whether all such molecules are merely predictions. Sequence conservation alone cannot establish conserved secretion or targeting. Protein effectors can also recruit defense proteins to processing bodies or alter nuclear trafficking (Liu et al., 2025b; Zhang et al., 2026b). These mechanisms regulate RNA-associated processes but are distinct from delivered regulatory RNAs.

Molecular origin also matters in vector systems. Cucumber mosaic virus (CMV) 2 b is virus encoded and binds JAZ proteins to alter defense and vector attraction (Wu et al. 2017). By contrast, salivary GAPDH and leafhopper vitellogenin are insect proteins that viruses exploit during infection or transmission (Wang et al., 2024a; Wang et al. 2024b). These observations describe different agents of manipulation and different fitness endpoints. A change favoring virus transmission need not increase vector fecundity, and an insect protein aiding transmission need not function primarily as an immune effector. Such cases delimit the review's scope while showing why crop evaluation must separate insect damage from pathogen spread.

## Feeding mode defines tissue exposure and immune bottlenecks

**Fig. 2:**
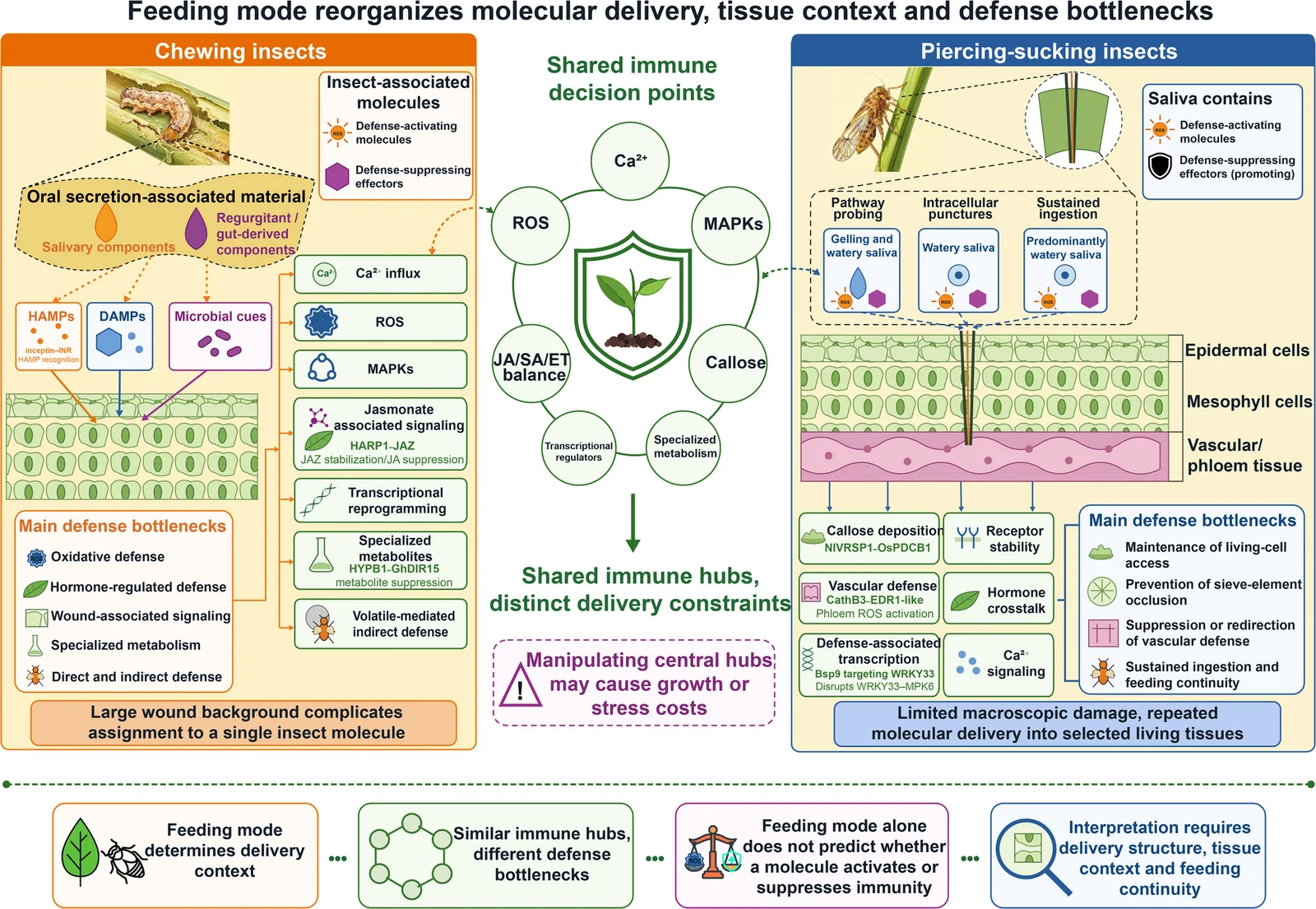
Feeding mode reorganizes molecular delivery, tissue context and defense bottlenecks. Chewing and piercing-sucking represent illustrative endpoints rather than an exhaustive classification. Gallers, miners and borers occupy additional insect positions, whereas spider mites are treated in the text as non-insect arthropod comparators with partly analogous feeding interfaces. Chewers release molecules into DAMP-rich wounds and primarily confront jasmonate-regulated, oxidative and indirect defenses. Stylet feeders deposit gelling saliva mainly during sheath formation and watery saliva during intracellular punctures and sustained ingestion, confronting callose, sieve-element occlusion, redox changes and vascular immunity. Representative molecule–bottleneck relationships are indicated adjacent to the corresponding defense processes: inceptin–INR, HAMP recognition; HARP1–JAZ, JAZ stabilization and jasmonate suppression; HYPB1–GhDIR15, specialized-metabolite suppression; NlVRSP1–OsPDCB1, disruption of callose deposition; CathB3–EDR1-like, phloem ROS activation; and Bsp9 targeting WRKY33, disruption of the WRKY33–MPK6 module and defense-associated transcription. Ca^2^⁺, calcium ion; DAMP, damage-associated molecular pattern; EDR1, enhanced disease resistance 1; ET, ethylene; GhDIR15, cotton dirigent protein 15; HAMP, herbivore-associated molecular pattern; INR, inceptin receptor; JA, jasmonic acid; JAZ, jasmonate ZIM-domain; MPK6, mitogen-activated protein kinase 6; OsPDCB1, rice plasmodesmata callose-binding protein 1; MAPKs, mitogen-activated protein kinases; ROS, reactive oxygen species; SA, salicylic acid. Regurgitant is a component of oral secretions, not a mutually exclusive release route. The salivation phases are schematic and do not imply a universal watery–gelling–watery sequence or fixed early/late assignments for individual proteins. Bsp9, whitefly salivary effector 9; CathB3, aphid cathepsin B3; HARP1 and HYPB1, cotton bollworm effector identifiers; NlVRSP1, planthopper salivary effector targeting OsPDCB1; WRKY33, WRKY transcription factor 33

Chewing and piercing**-**sucking in Fig. 2 are illustrative endpoints, not a complete classification of herbivory. Gallers, miners, borers and mites occupy intermediate or partly orthogonal positions along gradients of tissue disruption, protected feeding and maintenance of living cells. At the chewing endpoint, insects deliver molecules into a DAMP-rich wound where oxidases, proteases and plant enzymes rapidly modify the secretion. Major constraints include jasmonate-dependent toxins and antinutritive proteins, ROS, glucosinolate activation and volatile-mediated recruitment of natural enemies. *Arabidopsis* can perceive caterpillar oral secretions to enhance ROS-dependent glucosinolate hydrolysis, and herbivore-derived fatty-acid amides elicit early oxidative bursts (Block et al. 2018; Chen et al. 2025). The mechanistic challenge is to separate the activity of an insect molecule from responses caused by tissue disruption or microbial contaminants.

Chewers counter these defenses through several independent routes. Caterpillar effectors impair JA signaling, close stomata and reduce volatile release, or alter specialized metabolism (Lin et al. 2021). Cotton bollworm HYPB1 targets GhDIR15 and suppresses secondary-metabolite biosynthesis, whereas maize regulators ZmPP2C45 and ZmBELL4 reveal host nodes that limit benzoxazinoid defense (Wang et al., 2026c; Zhang et al., 2025a). Fall armyworm Sfapyrase instead activates defense, confirming that delivery route does not predict function (Yao et al. 2025). A useful comparison asks which plant bottleneck is altered—redox, hormone signaling, stomatal release or metabolite production—and whether the change improves insect performance.

Sustained phloem feeders must retain access to functioning vascular tissue. Some aphid salivary proteases degrade sieve-tube proteins (Furch et al. 2015). CathB3 acts through a different route: its propeptide bound tobacco EDR1-like, promoted phloem ROS, and limited a non-tobacco-adapted aphid lineage. CathB3 knockdown improved that lineage's performance (Guo et al. 2020). Thus, belonging to an enzyme class does not predict whether catalytic suppression or recognition-associated defense dominates. Host adaptation can involve reduced exposure to a detrimental salivary component, not only stronger suppressors. Planthopper endo-β−1,4-glucanase and salivary DNase II provide further distinct access- and defense-associated activities (Huang et al. 2019; Ji et al. 2017). The barrier affected, rather than the shared endpoint of reduced ingestion, identifies the relevant intervention.

The distinction between chewing and stylet feeding is a hypothesis about exposure, not a substitute for insect identity. Aphids, whiteflies and planthoppers differ in host range and associated organisms (Morin et al. 2024); aphid symbionts can also alter salivary-gland expression and plant defense (Wang et al. 2020). Thus, an apparent guild effect may instead reflect crop anatomy, host genotype or symbiont status. Within a compatible host, matched genotypes and feeding stages can test these alternatives. Across host-specialized insects, each natural host–insect pair needs its own controls, followed by comparison of homologous outputs; forcing every insect onto one plant genotype would introduce non-host stress. Exposure should predict which barrier is encountered, whereas the identity and direction of manipulation remain molecule-specific.

Spider mites are arachnids, not insects, and are included here as cross-arthropod comparators. Spider-mite salivary proteins suppress defense and promote reproduction in *N. benthamiana*, while Te16 and other proteins can elicit ROS, callose and jasmonate-associated responses (Cui et al. 2024; Villarroel et al. 2016). SHOT-family expression changes rapidly with host transfer, suggesting that broad host range can be supported by plastic secretion rather than a single universal effector set. The parallels indicate convergence on plant immune hubs despite independent salivary systems. Candidate secretion nevertheless remains distinct from demonstrated effector function.

Oviposition is another spatially distinct challenge. Plant responses to Pieris brassicae eggs share features with pattern-triggered immunity, indicating that immune surveillance begins before larval feeding (Gouhier-Darimont et al. 2013). Egg-associated signals can prime local and systemic defense, alter parasitoid recruitment and condition the later wound response. Including oviposition in the recognition–manipulation framework prevents an artificial focus on saliva and highlights that plants integrate sequential life-stage cues. The responsible ligands and receptors remain less resolved than those for inceptin, making this area suitable for receptor-discovery approaches guided by natural genetic variation. Feeding and oviposition define where signals arise. Their consequences depend on how local signals propagate, interact and terminate across the plant.

## Local perception scales into whole-plant defense and recovery

Once a signal is perceived at these distinct interfaces, calcium and protein phosphorylation provide the earliest opportunity to distinguish its spatial and temporal origin. Calcium signatures differ in amplitude, duration and cellular origin, and they are decoded by calcium-dependent protein kinases, calmodulin-like proteins and ion channels. CML42 coordinates *Arabidopsis* responses to *Spodoptera* herbivory and abiotic stress, while these kinases connect local attack to systemic defense (Romeis and Herde 2014; Vadassery et al. 2012). The same pathways operate in pathogen and abiotic responses, so a change in cytosolic calcium is not diagnostic of HAMP recognition. A mechanistic account connects the signature to a defined channel or sensor and then to an insect phenotype. Genetically encoded calcium reporters in epidermal, mesophyll and phloem-associated cells could reveal whether chewing and stylet feeding generate distinct spatial codes.

Mitogen-activated protein kinase studies provide a counterexample to treating stronger signaling as stronger resistance. Silencing *SIPK* and *WIPK* in *N. attenuata* did not simply increase herbivore susceptibility. In a separate study, MPK4 suppression revealed a JA-independent defense against the specialist *Manduca sexta*, but the same intervention did not protect against the generalist *Spodoptera littoralis* (Hettenhausen et al. 2013; Meldau et al. 2009). The comparison challenges both a linear kinase-to-resistance model and a guild-wide interpretation. Compensation by other pathways, herbivore sensitivity to different metabolites and altered host growth can each change the phenotype. Tests should therefore distinguish the kinase's immediate substrates from secondary physiological effects, using complementation and matched attacker comparisons. The relevant engineering target may be a particular substrate or metabolite branch, not the kinase itself.

JA is central to many antiherbivore responses, yet its effect is partitioned among biosynthetic branches, receptor complexes and downstream transcription factors. OsJAR1 controls herbivory-induced jasmonoyl-L-isoleucine accumulation in rice, and herbivore-induced jasmonate bursts can transiently reduce root growth (Fukumoto et al. 2013; Hummel et al. 2009). Ethylene can either cooperate with jasmonate or suppress particular outputs, including volatile production in rice (Mujiono et al. 2020). Pathway modularity explains why an effector that stabilizes JAZ repressors can strongly alter vector attraction without uniformly eliminating every defense. For crop engineering, the useful target is the branch that limits the pest, not a constitutive increase in total jasmonate.

Bt56 and Armet discriminate between a hormone response and its fitness consequence. Bt56-associated SA signaling accompanied improved whitefly performance. By contrast, Armet delivery increased SA accumulation and bacterial resistance without altering aphid survival or reproduction in the tested assays (Cui et al. 2019; Xu et al. 2019). These outcomes do not support a general rule that SA activation benefits sap feeders. They also leave open whether an Armet benefit requires a different dose, feeding-associated cofactor or ecological context; absence of an effect in one assay is not proof of no natural function. PAD4's separable roles against aphids and pathogens further weaken the inference from a pathogen marker to insect resistance (Louis et al. 2012). The decisive comparison is insect performance with and without the implicated hormone branch, under matched natural delivery. Marker induction can identify a response, but cannot establish either adaptive manipulation or crop protection.

Specialized metabolites convert signaling decisions into direct resistance. Cowpea ATP synthase fragments act as elicitors, but the protective output depends on each plant's chemical repertoire. Caffeic-acid O-methyltransferase-dependent flavonoid defenses contribute to sorghum resistance to fall armyworm, whereas herbivory-induced phenylacetaldoxime glucoside was characterized in tococa. These examples illustrate distinct plant chemical repertoires rather than one shared maize–sorghum pathway (Kundu et al. 2025b; Müller et al. 2024). Host regulators such as ZmPP2C45 and ZmBELL4 suppress biochemical defense, and cotton bollworm HYPB1 targets a dirigent protein to reduce secondary-metabolite biosynthesis. These mechanisms provide crop-specific intervention points that may carry fewer pleiotropic costs than manipulating central hormones.

Volatile emission links cell-autonomous immunity to ecological defense. Herbivore-induced terpenes vary among Arabidopsis accessions because of allelic and subcellular differences in terpene synthases (Huang et al. 2010). Stomatal conductance, carbon assimilation and oxylipin signaling can also change after herbivore perception, altering the quantity and timing of volatile release (Meza-Canales et al. 2017). A salivary effector that closes stomata and suppresses volatile emission demonstrates that insects can attack this output directly (Lin et al. 2021). Because volatiles may recruit natural enemies, repel herbivores or attract vectors, measurements in the relevant community and atmospheric context are necessary; emission intensity alone is not informative enough.

Local feeding can prime systemic tissues. Mythimna separata herbivory increases resistance in systemic maize leaves, while HERBIVORE ELICITOR-REGULATED1 links early perception to abscisic-acid accumulation and defense in *N. attenuata* (Dinh et al. 2013; Malook et al. 2021). Systemic responses can protect new growth, but they also redistribute carbon and may compromise tolerance to other stresses. Time-resolved assays can separate systemic acquired resistance, priming and resource reallocation. A primed leaf that responds faster after a second attack is mechanistically different from a leaf that already expresses costly defenses before challenge. Their distinction depends on experiments that close the chain from delivery and perception to behavior and whole-plant outcome.

## Distinguishing causal mechanisms from assay effects

**Fig. 3:**
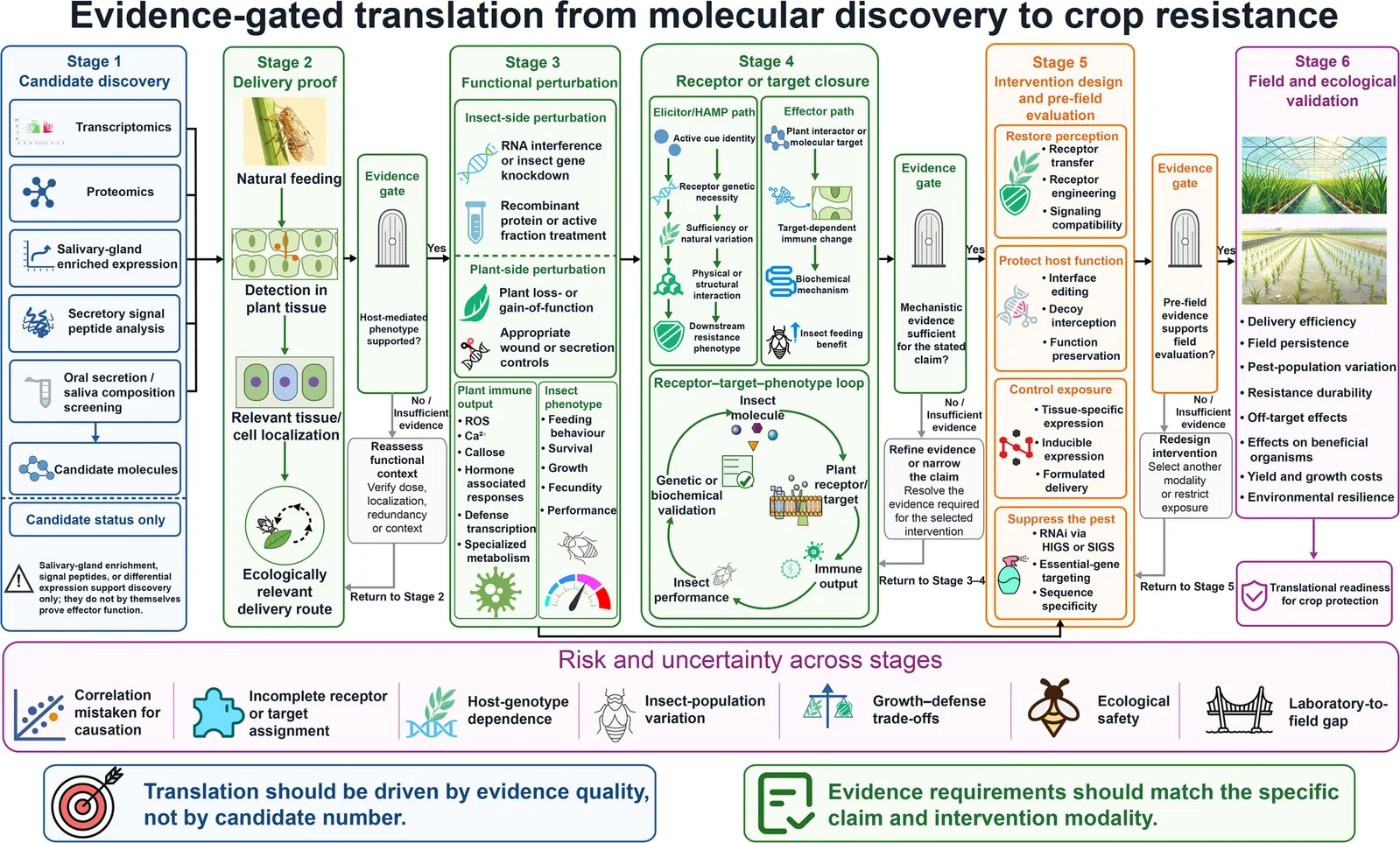
Evidence-gated translation from molecular discovery to crop resistance. The six stages shown are discovery, delivery proof, functional perturbation, receptor or target closure, intervention design and pre-field evaluation, and field and ecological validation. The workflow evaluates natural delivery and uses evidence gates to assess the host-mediated phenotype, whether mechanistic evidence is sufficient for the stated claim, and whether pre-field evidence supports field evaluation. Arrows marked “Yes” indicate forward progression, whereas “No / Insufficient evidence” outcomes initiate reassessment or redesign. Failure to demonstrate a host-mediated phenotype returns it to Stage 2 for evaluation of dose, localization, redundancy or biological context; incomplete mechanistic evidence for the stated claim returns it to Stages 3–4 for refinement of receptor or target assignment and causal variants; insufficient pre-field evidence returns it to Stage 5 for selection of an alternative intervention modality, improvement of delivery or restriction of exposure. An independent branch links insect-side functional evidence from Stage 3 to “Suppress the pest” in Stage 5 for RNAi-based strategies; this route bypasses the plant receptor or host-target mechanism gate but still requires evaluation of intervention efficacy, delivery feasibility, non-target risk and field performance. A failed gate therefore redirects the specific claim or application under test rather than demonstrating that the candidate lacks biological function. Stage 5 distinguishes restoring perception, protecting host function, suppressing the pest and controlling exposure. Stage numbers describe workflow progression rather than cumulative evidence grades or the independent questions evaluated in Table 3. Ca^2^⁺, calcium ion; HAMP, herbivore-associated molecular pattern; HIGS, host-induced gene silencing; RNAi, RNA interference; ROS, reactive oxygen species; SIGS, spray-induced gene silencing. Decision gates evaluate evidence generated by the relevant tests: delivery after Stage 2, host-mediated phenotype after Stage 3, and claim-specific mechanistic evidence after Stage 4. Their interpretation is claim-specific; complete receptor or target identification is not an absolute prerequisite for testing insect-directed RNAi efficacy

Figure 3 presents an experimental workflow, whereas Table 3 defines six independent questions: discovery, delivery, activity, receptor or target engagement, organismal phenotype and ecological context. These questions are not cumulative grades. Strong target biochemistry can coexist with uncertainty about native dose, and a plant-derived ligand does not require evidence of an insect-encoded origin. Secretomics supports discovery, whereas gland enrichment and artificial-diet detection differ from detection in attacked tissue (Boulain et al. 2018; Jonckheere et al. 2016). Each result should be recorded as demonstrated in the stated assay, unresolved, or not applicable to the claim. A negative result from an insensitive assay is not a functional test. This structure preserves useful findings while preventing strength in one question from masking a missing mechanistic link.

**Table 3:** Independent questions for mechanistic and translational claims. The rows are not cumulative grades and do not correspond one-to-one with the workflow stages in Fig. 3. Evidence in one row does not resolve another; not applicable must be distinguished from unresolved. Molecular termination, growth recovery and yield protection should be evaluated as distinct outcomes

Evidence domain	Question and informative evidence	Typical overclaim	Interpretation and decision consequence
Discovery	Is the candidate associated with secretion or feeding? Gland enrichment, sequence features and secretome detection prioritize tests	Treating predicted secretion as proof of delivery or effector function	Supports selection for study, not immune function; negative detection requires assay-sensitivity information
Delivery	Does the active molecule reach the proposed host compartment? Natural-feeding detection with contamination, localization and dose controls	Inferring activity merely from presence at the feeding interface	Establishes exposure; artificial-diet secretion alone does not establish plant entry. Record ligand origin and processing separately
Activity	Does the molecule alter a defined host output? Dose series, inactive variants, wound controls and gain/loss assays with matched exposure	Equating ectopic overexpression or toxicity with native function	Defines an activity in its assay context; distinguish defense activation, immune suppression and nonspecific stress
Engagement	Is the proposed receptor, target or substrate causal? Binding or catalysis, host genetics, interaction-deficient controls and complementation	Calling co-localization, co-receptor dependence or screening hits mechanistic closure	Supports molecular mechanism; enzyme/substrate and RNA/target-site tests need not use the same criteria as protein receptors
Organismal phenotype	Does the host-mediated effect alter insect performance or plant injury? Reciprocal perturbation where feasible, diet controls, rescue and baseline-matched epistasis	Inferring biological relevance from a one-sided perturbation	Tests biological relevance; insect-gene perturbation is not applicable to the biosynthetic origin of endogenous plant ligands
Ecological/agronomic context	Does protection persist across the intended host backgrounds, pest populations and environments, without unacceptable yield or non-target costs?	Generalizing from one genotype, population or controlled environment	Defines the tested deployment range, not universal durability. Measure signal decay, growth recovery and yield separately

Spatial technologies can determine whether a candidate reaches the cell in which its proposed target resides. Immunogold labeling, proximity labeling, split-fluorescent reporters and targeted mass spectrometry can complement conventional confocal imaging. For stylet feeders, combining these methods with electrical penetration graph phases allows localization to be assigned to pathway salivation, sieve-element puncture or sustained ingestion (Mugford et al. 2016). For chewers, fluorescently labeled purified proteins can be applied with defined wounds and tracked across the apoplast and endocytic compartments (Yan et al. 2023). Controls for tag cleavage, overexpression and tissue leakage matter because localization errors propagate directly into incorrect target models.

Structural and quantitative biochemistry are becoming necessary for receptor and target closure. Surface plasmon resonance, microscale thermophoresis and isothermal titration calorimetry can establish affinity, but the relevant binding test uses the processed molecule present during feeding. Cryo-electron microscopy or crystallography can then identify residues that discriminate recognition from virulence. Direct BISP–BPH14 binding provides closure for receptor engagement (Guo et al. 2023). Structural analysis of HARP1–JAZ repression provides a complementary example of effector–target closure (Zhang et al., 2026a). Structure-guided mutations create decisive controls when a variant retains stability and delivery but loses binding, allowing the interaction to be tested against both plant and insect phenotypes.

Dual-species genetics is most informative when it tests an alternative explanation, not merely when both organisms have been perturbed. Insect knockdown can impair secretion, development or nutrition before affecting the plant; stable plant expression can still distort dose and localization (Bos et al. 2010; Pitino and Hogenhout 2013). A feeding defect on artificial diet as well as on plants points toward an insect-intrinsic requirement, whereas a plant-specific defect supports host dependence. Loss of the defect in a plant target mutant is consistent with a shared pathway, but altered baseline susceptibility or a ceiling effect can produce the same pattern. Complementation, binding-deficient variants that retain stability, and matched exposure are needed to resolve these alternatives. Rescue and epistasis strengthen a mechanistic inference only when the rescue itself does not bypass the proposed route.

Behavioral phenotyping needs comparable molecular resolution. Electrical penetration graphs can identify whether an effector affects epidermal probing, xylem drinking, phloem salivation or ingestion (Zhang et al. 2022). Automated video and acoustic monitoring can quantify chewing bouts, while fluorescent tracers reveal vascular access. Behavioral measurements are most useful when synchronized with rapid plant reporters for calcium, ROS, pH and hormone activity. Rapid intracellular acidification during brown planthopper attack, for example, reveals an early defense state that the insect counters (Jiang et al. 2024). Synchronizing behavioral and plant signals can distinguish a primary molecular defect from secondary starvation or developmental decline.

Population genomics can identify variation relevant to durability, but sequence association does not establish the mechanism of escape. Hessian fly avirulence mapping and effector-family expansion illustrate distinct evidence: linkage to resistance specificity versus the available repertoire of candidate molecules (Aggarwal et al. 2014; Zhao et al. 2015). Matched surveys of insect effector variants and host resistance alleles can prioritize interactions for functional testing. Geography, population structure and host history must be accounted for, because they can associate an effector allele with virulence without that allele being causal. Testing natural variants at matched delivery, followed by insect challenge in the relevant host background, connects the population signal back to mechanism.

Field translation requires phenotyping that preserves mechanism. Near-isogenic lines or edited alleles can be challenged with multiple insect populations under realistic temperature, water and community conditions (Jing et al. 2017; Züst and Agrawal 2016). Pest abundance is interpreted alongside yield, recovery and beneficial-arthropod responses. Molecular biomarkers—such as effector delivery, receptor activation or target stabilization—can distinguish failure of the intended mechanism from poor expression or exposure. Iterative modeling can then combine allele frequency, fitness cost and landscape deployment to compare pyramids, mixtures and inducible strategies before large-scale release. Because field efficacy is not equivalent to agronomic benefit, mechanism-preserving validation also includes explicit measures of resistance, tolerance and productivity.

## Balancing resistance, tolerance and productivity

Resistance reduces herbivore success or injury; tolerance preserves plant performance at a given injury burden. These traits require different comparisons (Züst and Agrawal 2016). Equal numbers of insects do not ensure equal damage, and equal final yield does not establish equal physiological cost. Crop–aphid systems combine induced defense, constitutive barriers and compensation (Mou et al. 2023), so tolerance should be assessed across a range of measured feeding burdens, with uninfested controls defining baseline productivity. A line with fewer insects but a lower uninfested yield may offer little net benefit; one maintaining yield despite feeding may be useful but still support pest multiplication. Measuring both damage–yield relationships and insect population growth makes that trade-off visible.

Early immune signaling rapidly changes plant carbon balance. Herbivore perception can decrease photosynthetic carbon assimilation and stomatal conductance, while jasmonate bursts transiently reduce root growth (Hummel et al. 2009; Meza-Canales et al. 2017). Such responses may redirect resources toward defense, but they can also reduce the capacity to replace damaged tissue. The cost depends on attack duration, developmental stage and source–sink organization. Leaf defense markers can overestimate agronomic benefit when whole-plant carbon flux is not measured. Isotopic tracing and repeated imaging could determine whether a candidate resistance allele protects yield by reducing feeding, maintaining photosynthesis or accelerating post-attack recovery.

Hormone pathways also control compensation. Cytokinin perception modulates herbivory-induced signaling and defense in *N. attenuata*, whereas systemin-like peptides do not always reproduce the defensive role inferred from tomato (Schäfer et al. 2015; Schmidt and Baldwin 2006). The species-level variation cautions against transferring a growth regulator between species without testing developmental consequences. Tissue-specific or inducible manipulation may be preferable to constitutive activation because it preserves meristem function and reproduction outside the attacked zone. An effective intervention protects vulnerable tissue, maintains source–sink coordination and decays after feeding stops.

Compensation can be exploited by insects. Competition among herbivores can produce hyper-suppression of plant defense and overcompensation of herbivore reproduction, meaning that reduced damage by one attacker may increase the success of another (Schimmel et al. 2017). Plant viruses and symbionts can further alter source–sink relations and vector behavior (Wang et al. 2020). Tolerance breeding also accounts for community effects because yield preservation under a single pest may not persist during co-infestation. Factorial field experiments with dominant pest combinations can identify whether a molecular resistance mechanism remains additive, becomes redundant or creates an ecological release for another herbivore.

The BISP system already provides experimental evidence of a resistance–cost trade-off. Constitutive BISP expression in a Bph14 background improved resistance during prolonged infestation but reduced growth and yield. In a separate comparison, OsNBR1 loss slowed BISP clearance, prolonged defense outputs after insect removal and increased resistance during 14 days of infestation (Guo et al. 2023). These findings argue against optimizing either activation or clearance in isolation. The yield cost of persistent ectopic BISP is not a direct estimate of the yield benefit from natural post-attack clearance, and increased resistance in the knockout does not establish its agronomic superiority. Thus, self-regulation has a stronger empirical basis than a generic growth–defense hypothesis (Wu et al. 2024), while the optimum under repeated, intermittent attack remains unresolved.

Productivity remains the final translational endpoint. Increasing flavonoid defenses, annexin abundance or specialized-metabolite pathways can reduce pest performance (Su et al. 2025; Zhang et al., 2025b). However, each intervention may also alter plant nutrition, development or responses to drought and pathogens. Multi-environment trials compare yield stability, quality traits and recovery with pest suppression. Mechanistic biomarkers can explain trade-offs: receptor activation measures perception, target occupancy measures effector control and metabolic profiling measures defense allocation. Combined, the three measurements can identify interventions that provide sufficient resistance at lower physiological cost than the strongest laboratory phenotype. Even a low-cost intervention, however, may be short-lived if insect populations escape, so agronomic value is inseparable from evolutionary persistence.

## Host specialization and effector evolution shape durability

Effector repertoires evolve at several scales, linking molecular mechanism directly to resistance durability. Gene-family expansion creates raw diversity, positive selection changes binding surfaces, and expression plasticity changes which molecules are delivered on a particular host. Aphid salivary effector families show lineage-specific expansion and rapid evolution, while C002, PIntO1 and PIntO2 promote colonization in a plant-species-specific manner (Boulain et al. 2018; Pitino and Hogenhout 2013). The pattern argues against assuming that an effector validated in one aphid–host pair will retain the same target or phenotype in another. Comparative functional assays belong alongside sequence analyses.

Host-dependent transcription provides a faster response than sequence evolution. SHOT genes are induced after transfer to particular hosts and downregulated more slowly after return to a non-inducing host. Aphid biotypes likewise show differential expression of candidate salivary effectors on distinct host plants (Boulain et al. 2019). Expression plasticity can generate apparent genotype-by-host interactions without changes in coding sequence (Jonckheere et al. 2018). Resistance screening is further complicated because insects reared for generations on one host may deliver a different salivary mixture from newly transferred field populations.

Target specificity provides another route to specialization. The VPS52-targeting aphid effector acts in a host-dependent manner, and Me10 engages a tomato 14–3-3 protein associated with resistance to non-adapted aphids (Chaudhary et al. 2019; Rodriguez et al. 2017). The host-specific interactions suggest that non-host resistance can arise when an effector fails to manipulate the available target or instead exposes the insect to surveillance. Mapping natural variation on both sides of the interface can identify target residues that preserve plant function but reduce effector binding. Such alleles may cost less than constitutive defense activation.

Resistance genes impose strong selection on insect populations. The Hessian fly–wheat system provides a clear genetic example of a gene-for-gene interaction between an insect and its host plant (Stuart 2015). Individual wheat H resistance genes correspond to specific insect avirulence loci. The H13–vH13 pair currently provides the strongest functional evidence. High-resolution mapping identified vH13 as a secreted salivary effector gene, while virulence-associated insertions were linked to loss of its transcript. RNA interference (RNAi) of *vH13* partially enabled otherwise avirulent larvae to survive on *H13*-containing wheat, supporting a causal role for this effector in *H13*-mediated resistance (Aggarwal et al. 2014). A candidate vH24 effector encoding a secreted protein phosphatase 2 C has likewise been genetically associated with *H24*-specific avirulence, although its functional validation remains less complete (Zhao et al. 2016). The combined genetic and RNAi data provide causal evidence for vH13 involvement and strong genetic correspondence at other loci, but direct physical recognition by the corresponding H proteins has not been demonstrated. Durable deployment depends on resistance-gene combinations and surveillance of avirulence alleles; damage scores alone are inadequate.

Comparisons of brown planthopper populations reveal physiological routes around resistance but require caution about causality. The Bph1-virulent Mudgo-BPH population sustained longer phloem ingestion, produced more honeydew and gained more weight than TN1-BPH on Mudgo rice; transcriptomic and biochemical differences accompanied those phenotypes (Wan et al. 2019). These findings establish population-level compatibility with a resistant host. They do not establish that every differentially expressed digestive, metabolic or detoxification gene causes resistance breakdown: improved nutrition after successful feeding could generate many of the same changes. Reciprocal transfer, early sampling before feeding diverges, and perturbation in matched rice backgrounds can distinguish a cause of restored access from a consequence of it.

Salivary-gland studies identified population differences in putatively secreted genes and host-dependent changes in sheath, metabolic and calcium-associated components (Ji et al. 2013; Wang et al. 2015b). In BPH15-associated material, silencing *NlCPR* or *CYP4C61* impaired feeding on resistant rice (Peng et al. 2017); a genome-wide survey identified 644 carbohydrate-active enzymes and genotype-responsive candidates (Liu et al., 2025a). The evidence is heterogeneous: differential expression supports association, whereas perturbation supports a functional requirement but can still reflect general physiological impairment. Neither establishes an avirulence determinant for a particular resistance gene. Near-isogenic rice comparisons and explicit susceptible-host controls are therefore needed before these genes are assigned to recognition escape, vascular access or metabolic compensation (Ye et al. 2024).

Three competing explanations make the next experiments more discriminating. Recognition escape predicts reduced activation by a virulent population's effector at matched delivery in a plant carrying the cognate receptor. Restoration of vascular access predicts sustained ingestion despite preserved recognition. Metabolic tolerance predicts improved insect performance despite comparable exposure to host defenses. These possibilities are not mutually exclusive, but they imply different measurements and deployment strategies. BISP defines a tractable recognition assay for BPH14, whereas current Bph1- and BPH15-associated population studies largely resolve feeding and physiological adaptation (Guo et al. 2023; Peng et al. 2017; Ye et al. 2024). Plant genotype-by-insect population experiments that combine delivered protein, early immune output, electrical penetration graphs and subsequent growth can assign the dominant failure route. Without this partition, adding another resistance gene may reproduce the vulnerability already bypassed.

*NLR* deployment must therefore address correlated failure as well as individual gene effectiveness (Boissot 2023). Two receptors can recognize different molecules yet depend on the same downstream component or fail against the same virulent population. Conversely, a receptor and a vascular defense allele may impose distinct barriers, but their combination could increase growth costs. BISP-regulated signaling supplies one candidate mechanism for controlling duration (Guo et al. 2023); it does not establish compatibility with every second resistance trait. Cross-challenge of single and combined alleles, with shared pest populations and uninfested controls, should determine whether the combination adds protection, duplicates an existing barrier or introduces a new cost.

Ecological context can reverse molecular outcomes. Competition among herbivores can cause overcompensation through hyper-suppression of plant defense, and parasitoid-associated viruses alter plant responses indirectly through their caterpillar hosts (Schimmel et al. 2017). Virus infection can change vector preference through both plant volatiles and the insect olfactory system (Ai et al. 2025). The ecological interactions imply that resistance measured against a single insect genotype in isolation may fail in communities containing competitors, natural enemies or pathogens. Factorial experiments that preserve the most influential ecological partners provide the next step beyond pairwise assays. Evolutionary and ecological constraints define the boundary conditions for translating closed mechanisms into crop protection.

## Mechanism-guided resistance design and deployment

Resistance design should begin with the biological step that limits protection rather than with a preferred technology (Fig. 3). Absence of perception favors transfer or engineering of a receptor when the relevant insect-associated cue is naturally delivered and the recipient crop provides compatible signaling components. Exploitation of a defined plant protein favors selective editing of the effector–target interface, decoy-based interception or another antivirulence strategy, whereas an accessible and sufficiently conserved insect-essential gene favors RNAi. When constitutive protection imposes a growth cost, tissue-specific or herbivore-inducible deployment may be preferable. Each option must then be evaluated against three additional constraints: whether the intervention can reach the relevant feeding interface, whether protection persists across pest populations, and whether agronomic or non-target costs remain acceptable. Inceptin–INR, BPH14-mediated recognition and the resistance-associated OsPDCB1 haplotype illustrate three starting points: restoring perception, exploiting an existing receptor and protecting a vascular defense component. They do not define a universal hierarchy of readiness (Guo et al. 2023; Steinbrenner et al. 2020; Yang et al. 2026).

CRISPR-based editing of a plant susceptibility factor is justified only when the effector-associated interaction can be separated from the target’s endogenous function. Whenever possible, editing should alter the effector-binding surface or a relevant cis-regulatory element rather than disrupt the entire host gene. PUB33 and GhFKBP17-2 illustrate why this is demanding: the host proteins participate in ubiquitination or protein folding rather than existing solely as effector docking sites (Dong et al. 2023; Wang et al. 2024c). An edit that reduces binding but compromises normal clients may lower insect performance by weakening the plant. Candidate alleles should therefore retain biochemical activity, endogenous interaction partners and normal developmental function in complementation tests before advancing to insect and yield assays. Natural variation, structure-guided substitutions, base editing or prime editing may be preferable to gene knockout when they can reduce effector binding while preserving host activity. ZmPP2C45 and ZmBELL4 provide a different route through biochemical defense regulation (Zhang et al., 2025a), but altering such regulators is not equivalent to selectively removing an effector interaction. The intervention should not advance if resistance results from general physiological impairment, unacceptable yield loss or increased susceptibility to other stresses.

For insect-directed RNAi, identification of the plant target of an insect effector is desirable for mechanism interpretation but is not a prerequisite for pest suppression. Insect-side perturbation of NlMLP, Nlsp5, aphid MIF proteins and Armet produced feeding or survival phenotypes (Naessens et al. 2015; Qi et al. 2025; Shangguan et al. 2018; Wang et al., 2015a). These molecules are therefore candidate RNAi targets, but their practical value depends on exposure, sequence conservation and functional redundancy. Host-induced gene silencing (HIGS) can provide continuous exposure by expressing insect-targeting double-stranded RNA in the crop, whereas spray-induced gene silencing (SIGS) permits non-integrated and replaceable deployment. HIGS is favored when sustained protection and predictable tissue expression are required; SIGS is favored when target sequences may need to be reformulated as pest populations change or when stable transformation is undesirable. Neither route overcomes poor insect uptake, rapid degradation in the gut, compensatory paralogs or insufficient silencing in the relevant life stage. Plant-mediated RNAi against a cotton bollworm P450 provided an early proof of principle for reducing larval tolerance of gossypol (Mao et al. 2007). This is a delivery and susceptibility example, not evidence that the targeted P450 is a salivary effector.

Delivery should be matched to feeding biology. Phloem-restricted expression is most relevant to aphids and planthoppers, whereas wound-responsive expression may better expose chewing insects to protective RNA. For SIGS, naked RNA is vulnerable to nucleases, ultraviolet radiation and rain wash-off. Clay, lipid, polymer or other nanoparticle carriers may improve adhesion, persistence and uptake, but they are delivery platforms rather than independent resistance mechanisms. Their value should therefore be measured as increased bioavailable RNA or improved pest control at a lower applied dose, and not inferred solely from greater physical stability. Geographic variation in the target sequence, possible cross-silencing of beneficial arthropods and carrier-specific environmental effects must be examined before deployment. A direct example is foliar delivery of clay-associated dsRNA against whitefly, which improved protection against immature stages compared with naked dsRNA (Jain et al. 2022). This result supports testing formulation-dependent efficacy, not assuming that the same carrier will work across insect species.

Antivirulence approaches aim to preserve plant function while blocking exploitation. Increasing rice ANNEXIN expression counters NlAnnexin-like5, while preventing effector-associated NtRLP4 destabilization is another candidate route (Wang et al., 2026b; Zhang et al., 2025b). Their proposed durability advantage should not be assumed. The most selective version of this strategy would alter only the effector-sensitive feature of the host component. When direct editing is constrained by an essential endogenous function, an engineered decoy or target mimic could instead sequester the effector or convert its engagement into immune activation. Decoy performance would require sufficient expression in the naturally attacked tissue, higher effective affinity than the endogenous target and minimal interference with normal host complexes. Because these conditions have rarely been resolved in plant–insect systems, decoys should presently be treated as a testable design route rather than as an established resistance modality. Any intervention that reduces feeding or reproduction can favor insects that evade it, even without acute toxicity; escape could involve a different effector, altered dose or use of another host target. These approaches merit tests of physiological selectivity and fitness costs of escape. Lower direct lethality is not evidence of weaker selection, and a molecularly elegant intervention can still fail at the population level.

Receptor engineering addresses a different limitation: failure to perceive a naturally delivered molecule. Transfer of an established receptor such as INR provides a direct route when ligand exposure and downstream signaling are compatible. Chimeric pattern-recognition receptors or NLRs with engineered recognition domains could, in principle, extend the range of detectable insect molecules. Synthetic receptors must retain appropriate co-receptor recruitment, subcellular localization and activation thresholds in the recipient crop; constitutive or ligand-independent activity would convert resistance into autoimmunity. Expression control is therefore part of receptor design rather than an afterthought. Phloem-specific promoters may restrict protection to tissues attacked by sap feeders, whereas wound- or herbivore-inducible promoters may reduce the growth cost of constitutive defense. Their principal failure modes are promoter leakiness, activation that occurs only after substantial feeding damage, and insufficient expression in the cells receiving the insect molecule. Synthetic-receptor and inducible-expression strategies remain less mature in plant–insect systems than conventional resistance-gene deployment and require crop-specific validation.

Elicitor-based protection shares the delivery challenge of SIGS: the active molecule must reach the appropriate tissue at an effective and tolerable dose. Plant peptides and endogenous metabolites such as loliolide can induce resistance-associated responses (Murata et al. 2019; Shen et al. 2022), but activity after infiltration alone is insufficient to predict practical efficacy. Stability on leaves, access to the receptor and timing relative to attack determine whether the laboratory mechanism is engaged. Encapsulation or nanoparticle association may extend persistence or facilitate entry, but the formulation must preserve ligand activity and should not itself induce stress responses that are mistaken for receptor-specific protection. Treatment-by-infestation comparisons should include uninfested plants to reveal growth costs and repeated attacks to test persistence. An intervention should advance because it reduces injury or preserves yield at a feasible dose, not because it maximizes a marker transcript.

Indirect defense can complement direct resistance. Receptor-mediated caterpillar detection can recruit predators, and plant elicitor peptides regulate volatile-based tritrophic interactions (Guayazán Palacios et al. 2026a; Huffaker et al. 2013). Volatile deployment is environment dependent and may attract herbivores or vectors under some conditions (Wu et al. 2017). Viral manipulation of JAZ proteins shows how the signaling network can be co-opted to increase vector attraction. Field tests can measure natural-enemy recruitment, vector behavior and pathogen transmission together. Volatile emission is not intrinsically beneficial.

Regulatory and biosafety requirements differ among intervention classes and jurisdictions. Risk assessment of gene-edited crops should consider characterization of the intended allele, possible off-target changes, trait stability, pleiotropic effects and gene flow. Regulatory treatment varies among jurisdictions and may also differ according to whether the edit retains introduced DNA. Assessment of HIGS crops should additionally consider characterization of the expressed RNA sequence, abundance, tissue distribution and environmental exposure, whereas SIGS formulations may be regulated as externally applied biological pesticides. Sequence similarity provides an initial screen for RNAi specificity but cannot replace exposure-based testing. Pollinators, predators and parasitoids may encounter active RNA through plant tissue, pollen, honeydew, prey or hosts. Assessment should cover acute mortality as well as chronic and sublethal effects on development, fecundity, feeding and behavior. For nanoparticle formulations, the RNA or elicitor cargo and the carrier material should be evaluated independently. Relevant endpoints include degradation, persistence in soil or water, movement beyond the treated tissue and possible trophic transfer. As a jurisdiction-specific example, the US Environmental Protection Agency registered the externally applied RNAi active ingredient ledprona for Colorado potato beetle control in 2023 (EPA 2023). This precedent does not establish equivalent authorization or safety for other RNA sequences, formulations, crops or jurisdictions.

Durability requires a deployment hypothesis with an explicit and monitorable failure route. Hessian fly genetics and aphid resistance experience show why resistance must be considered against variable pest populations (Boissot 2023; Zhao et al. 2015). A pyramid is promising when its components have different experimentally demonstrated failure routes; counting genes or immune outputs is insufficient. Mixtures, rotations or inducible protection may alter selection and exposure, but their value depends on pest movement, local allele frequencies and agronomic practice. Monitoring should therefore connect deteriorating control to changes in effector sequence or delivery, host target function, and feeding behavior. This diagnosis determines whether to replace a recognition component, reinforce a vascular barrier or address a delivery problem. Molecular monitoring adds value only when it changes a management decision.

## Comparative synthesis: what the evidence changes

The comparisons support three current judgments, not simply a longer list of experiments. First, defense markers cannot rank the biological value of insect molecules. Bt56-associated SA activation accompanies whitefly benefit, whereas Armet-induced SA and pathogen resistance did not improve aphid performance in the tested assays (Cui et al. 2019; Xu et al. 2019). Those results are not a direct contradiction because the molecules, hosts and interventions differ; together, they reject a general rule that SA activation favors sap feeders. Mp10 supplies a within-system test: loss of the extra protection induced by OG can coexist with greater baseline resistance (Gravino et al. 2025). Consequently, an assay must state whether it measures pathway responsiveness or net resistance. Treating the two as equivalent would reverse the interpretation of a candidate's value.

Second, superficially similar insect-knockdown phenotypes have different causal support. MIF depletion impaired aphids on plants but not artificial diet. Pea-aphid RNAi affected ApMIF1–3, whereas the plant immune assays and rescue by plant expression concerned MpMIF1 in the green peach aphid system (Naessens et al. 2015). These controls support host dependence, but do not assign all family-level effects to ApMIF1 or identify the natural plant compartment from artificial-diet secretion. Armet likewise has an insect feeding requirement, yet its measured SA response remains unconnected to aphid benefit (Cui et al. 2019; Wang et al., 2015a). Sm9723 presents another gap: immune suppression was tested in *N. benthamiana*, whereas insect performance was tested on wheat (Zhang et al. 2022). This supports a candidate immune effector, not a demonstrated wheat immune mechanism. These cases support different levels of inference; a uniform label of either complete proof or missing host-dependence evidence would obscure those differences.

Third, the evidence supports regulated defense duration, not a monotonic benefit from either stronger activation or faster clearance. BISP persistence increased resistance under prolonged attack in the tested rice backgrounds, while constitutive BISP expression also imposed growth and yield costs (Guo et al. 2023). These observed outcomes make the trade-off concrete. They do not show that faster natural clearance necessarily restores yield or that an OsNBR1-deficient plant is agronomically preferable. The remaining uncertainty is the balance under realistic attack schedules, not whether persistent activation can carry a cost (Wu et al. 2024). Molecular termination, renewed growth and final yield must therefore remain distinct when this mechanism is used to justify crop improvement.

These judgments also delimit transferability. Host-specific elicitation and species-specific effector activity show that related biological outputs do not establish interchangeable molecules (Louis et al. 2013; Snoeck et al. 2022b). Accession-dependent responses demonstrate that the plant background can matter even within a species (Wu et al. 2008). Thus, recurrence of calcium signaling, hormone regulation or protein turnover supports convergence on host processes, but is insufficient to claim broad-spectrum efficacy for a particular target edit. Together, these comparisons show that translational value depends on matching a supported mechanism to the crop, feeding interface and limiting biological step.

## Limitations

This Review uses purposive case selection to compare mechanistic chains, so it cannot estimate how frequently any recognition or manipulation route occurs across herbivorous insects. Mechanistically rich rice–planthopper and solanaceous systems, protein effectors and positive findings are consequently overrepresented, whereas understudied taxa, non-protein signals, negative results and field-scale evidence are less visible. Because the search and selection were not systematic and study-level risk of bias was not formally assessed, relevant studies may have been missed and the balance of examples may reflect publication and model-system bias. Absence from the synthesis therefore carries no inference of biological absence. The cap on the initial working library, unverified completeness of the Web of Science export and targeted rather than comprehensive later additions further limit coverage.

Heterogeneity in host genotype, insect population, molecular delivery, dose, developmental stage, assay design and endpoints also prevents pooled effect-size estimation or direct ranking of mechanisms and interventions. Binding, defense-marker, feeding, fecundity, injury and yield outcomes close different parts of a causal chain and are not interchangeable. Accordingly, R and M coordinates indicate the highest level of mechanistic closure supported by current evidence; they do not measure molecular potency, prevalence, agronomic importance or field readiness. The map and associated judgments represent a time-specific qualitative synthesis and should be updated as natural-delivery, genetic, population and field evidence accumulates. These constraints define the framework’s appropriate use: diagnosing missing causal links, comparing the evidentiary basis of claims and prioritizing experiments, rather than predicting field performance or establishing a quantitative hierarchy of resistance strategies.

## Outstanding questions

Despite recent progress, the natural exposure conditions of many insect-associated molecules remain unresolved. Which molecular forms, doses and temporal profiles occur during distinct phases of natural feeding? How does the same molecule shift between plant recognition and host manipulation across plant genotypes and insect populations? Which receptor or target interactions causally connect natural delivery with insect performance, rather than merely correlating with defense markers? The combined effects of co-delivered molecules, microbial partners and functional redundancy also require resolution. It remains unclear how signal duration and termination influence resistance, growth recovery and yield under repeated infestation, and which resistance strategies remain effective across pest populations, environments and non-target communities.

## Priority actions

Research priorities should focus on closing the causal and translational gaps identified by this framework. Natural delivery should first be quantified through feeding-phase-resolved proteomics, spatial imaging and measurements of molecular dose. Causal chains should then be tested through reciprocal plant and insect perturbations, rescue experiments and binding-deficient molecular variants. Natural variation and structural information can guide the identification of receptor or target alleles that disrupt insect exploitation while preserving endogenous plant function. Experiments should also test biologically plausible combinations of co-delivered molecules instead of examining every salivary component in isolation. Resistance, post-attack growth recovery and yield should be measured separately under repeated or intermittent infestation. Finally, promising strategies should be evaluated across pest populations and environments for durability, monitorable failure routes and effects on beneficial arthropods.

## Conclusions

At the plant immune interface, a molecular response is not equivalent to a mechanism of resistance. Recognition can coexist with manipulation, feeding requirements may be independent of immune activity, and conserved host targets can generate different outcomes across plant and insect backgrounds. Informative experiments therefore preserve natural delivery and test alternative explanations for the observed phenotype. The six evidence questions should be evaluated independently: strong target biochemistry cannot resolve natural exposure, and plant-derived ligands do not require an insect-gene test. Each unresolved link points to a specific next experiment or design decision.

For agricultural translation, priority should go to interventions that match a defined mechanistic limitation, reach the relevant feeding interface and have a monitorable failure route. Candidate strategies should be evaluated across pest populations and repeated infestations for resistance, recovery, yield, regulatory feasibility, environmental persistence and effects on beneficial organisms. The recognition–manipulation framework therefore supports crop-specific decisions by separating what is mechanistically established from what remains to be tested.

## Data Availability

No data were used for the research described in the article.
